# Of the Nature and Treatment of Apthæ

**Published:** 1844-11

**Authors:** 


*Of the Nature and Treatment of Apthæ.*—Next to the induration of the cellular tissue, the disease which carries off the greatest number of newly-born children in the foundling hospitals is thrush. Hitherto we have been entirely ignorant of the cause and nature of this serious disease. The greater number of pathologists saw in apthæ only a pseudo-membranous production consecutive on

[*About 9 grains.]

an idiopathic inflammation. With others it was a symptomatic inflammation. Neither were medical men agreed as to its mode of transmission; some regarded it as contagious, while others formally denied that it was so. According to the researches of M. Gruby, thrush is produced by the development of a cryptogamic plant.

Apthæ present themselves in the form of white masses, covering the whole of the mucous membrane of the mouth, and extending sometimes into the pharynx, œsophagus, stomach, and small intestines. The commencement of the disease is characterized by small, conical, whitish elevations, twenty-five millimetres in diameter, dispersed over the mucous membrane of the mouth; these elevations soon increase in size, and extend rapidly in the form of a pseudo-membrane strongly adherent to the subjacent tissue, from two to three millimetres thick, and covering sometimes the whole extent of the alimentary canal. A portion of this substance, examined under the microscope, is found to be wholly composed of a collection of cryptogamic plants. The roots are implanted in the cellules of the epithelium; they are cylindrical, transparent, and about 1-480th of a millimetre in diameter; during the development they perforate the entire series of cellules which compose the epithelium, to arrive at the free surface of the mucous membrane. The stems, which spring from the surface of the epithelium, are equally transparent, are divided at certain distances by septa, and contain corpuscles in their interior; they are cylindrical, straight, about one fourth of a millimetre in length, and 1-400th of a millimetre in width; the stems are divided into branches, which are again subdivided, bifurcating at an acute angle. These branches are composed of very distinct oblong cellules, containing one, two or three round and transparent nuclei; their lateral parts have sporules here and there, and their ends especially have a great number. The diameter of these sporules is from 1-200th to 1-500th of a millimetre.

These cryptogamic plants have considerable analogy with the sporotrichium described by some botanists. As they are very fragile, they become detached by the movements of the organs lined by the mucous membrane, and becoming mingled with the food, are carried into the alimentary canal, of which they afterwards cover a considerable extent. Those children in whom this extension of disease takes place very largely, fall into marasmus, and soon die. As M. Gruby has constantly found in the white substance of apthæ only these plants and the cellules of the epithelium, and never any production of inflammation, he deems himself authorized to conclude that thrush is nothing else than a cryptogamic plant vegetating on the living mucous membrane.

M. Trousseau employs the following collutory successfully in the treatment of thrush:—One gramme of hydrochloric acid, ten grammes of honey. He also recommends the following application:—Equal parts of finely-powdered borax and honey mixed together.—*Med. Times*, from *Bouchardat's AinuarneThra-de ne peutique* for 1844, in *Am. Jour.*

